# Kaolin-induced chronic hydrocephalus accelerates amyloid deposition and vascular disease in transgenic rats expressing high levels of human APP

**DOI:** 10.1186/2045-8118-12-2

**Published:** 2015-01-24

**Authors:** Gerald D Silverberg, Miles C Miller, Crissey L Pascale, Ilias N Caralopoulos, Yuksel Agca, Cansu Agca, Edward G Stopa

**Affiliations:** Department of Neurosurgery, The Warren Alpert Medical School of Brown University and the Aldrich Laboratories at Rhode Island Hospital, 593 Eddy Street, Providence, RI 02903 USA; Department of Veterinary Pathobiology, University of Missouri College of Veterinary Medicine, Columbia, MO 65211 USA; Department of Pathology (Neuropathology), Warren Alpert Medical School of Brown University and the Aldrich Laboratories at Rhode Island Hospital, 593 Eddy Street, Providence, RI 02903 USA; Stanford University, 710 Frenchmans Rd, Stanford, CA 94305 USA

**Keywords:** Normal pressure hydrocephalus, Transgenic rat, Kaolin-induced hydrocephalus, Amyloid-beta peptide, Cerebrovascular disease, Amyloid angiopathy, Solute clearance, CSF circulation

## Abstract

**Background:**

Normal pressure hydrocephalus (NPH) is most common in the elderly and has a high co-morbidity with Alzheimer’s disease (AD) and cerebrovascular disease (CVD). To understand the relationship between NPH, AD and CVD, we investigated how chronic hydrocephalus impacts brain amyloid-beta peptide (Aβ) accumulation and vascular pathology in an AD transgenic rodent model. Previously we showed that the altered CSF physiology produced by kaolin-hydrocephalus in older wild-type Sprague–Dawley rats increased Aβ and hyperphosphorylated Tau (Silverberg et. al. *Brain Res.* 2010, 1317:286–296). We postulated that hydrocephalus would similarly affect an AD rat model.

**Methods:**

Thirty-five transgenic rats (tgAPP21) that express high levels of human APP and naturally overproduce Aβ40 were used. Six- (n = 7) and twelve-month-old (n = 9) rats had hydrocephalus induced by cisternal kaolin injection. We analyzed Aβ burden (Aβ40, Aβ42 and oligomeric Aβ) and vascular integrity (Masson trichrome and Verhoeff-Van Gieson) by immunohistochemistry and chemical staining at 10 weeks (n = 8) and 6 months (n = 5) post hydrocephalus induction. We also analyzed whether the vascular pathology seen in tgAPP21 rats, which develop amyloid angiopathy, was accelerated by hydrocephalus. Age-matched naïve and sham-operated tgAPP21 rats served as controls (n = 19).

**Results:**

In hydrocephalic tgAPP21 rats, compared to naïve and sham-operated controls, there was increased Aβ 40 and oligomeric Aβ in hippocampal and cortical neurons at 10 weeks and 6 months post-hydrocephalus induction. No dense-core amyloid plaques were seen, but diffuse Aβ immunoreactivity was evident in neurons. Vascular pathology was accelerated by the induction of hydrocephalus compared to controls. In the six-month-old rats, subtle degenerative changes were noted in vessel walls at 10 weeks post-kaolin, whereas at six months post-kaolin and in the 12-month-old hydrocephalic rats more pronounced amyloid angiopathic changes were seen, with frequent large areas of infarction noted.

**Conclusions:**

Kaolin-hydrocephalus can accelerate intraneuronal Aβ40 accumulation and vascular pathology in tgAPP21 rats. In addition, disrupted CSF production and reduced CSF turnover results in impaired Aβ clearance and accelerated vascular pathology in chronic hydrocephalus. The high co-morbidity seen in NPH, AD and CVD is likely not to be an age-related coincidence, but rather a convergence of pathologies related to diminished CSF clearance.

## Background

Normal pressure hydrocephalus (NPH) is a clinically-diagnosed disease that presents with one or several symptoms and signs, including gait disturbance, incontinence and dementia [1]. The gait disturbance is described as a gait apraxia (magnetic gait), and the urinary incontinence is often nocturnal. There is an associated ventricular enlargement, usually involving all ventricles, and some enlargement of the subarachnoid space (SAS) over the convexities. There is usually intermittent elevation of the cerebrospinal fluid (CSF) pressure most often at night and often during rapid eye movement (REM) sleep [2–4]. Clinical diagnosis rests on clinical and radiologic findings as well as the effects of large volume CSF removal [5].

Although there is no pathological finding that confirms the clinical diagnosis of NPH on brain biopsy or at post-mortem, there is often evidence for both Alzheimer’s disease (AD) and cerebrovascular disease (CVD) with a frequency that precludes the idea that these are unrelated afflictions of the elderly [6–9]. Indeed, initially these findings were thought to be simply unrelated coincidental diseases of the elderly, but that is no longer tenable.

In patients with the symptoms and signs of NPH, there is a very high co-morbidity with both AD and CVD. The incidence of AD pathology in patients with NPH is much higher than expected if the two diseases were unrelated diseases of advancing age. For instance, anywhere from 25% to 75% of NPH patients will have evidence of AD pathology, depending upon the degree of dementia, on brain biopsy at the time of shunt placement or at autopsy [10–12]. In aging, only 10% of subjects over 65 will have clinical or histological evidence of AD [13]. Evidence of CVD is similarly increased in NPH over normally aged subjects.

Alzheimer’s disease is characterized by amyloid plaques (neuritic or dense-core), composed of amyloid-beta peptides (Aβ) and neurofibrillary tangles made up of paired helical filaments of hyperphosphorylated tau protein (pTau). Amyloid angiopathy is also characteristic of AD brains. This vascular pathology is characterized by vascular deposition of Aβ [14, 15]. In recent years it has become accepted that the accumulation of Aβ in non-familial AD is due to an inability to clear these peptides from the brain, rather than an overproduction as seen in familial AD [16–18].

In previous reports, it was shown that both Aβ and pTau protein accumulated in older (12 months) wild-type Sprague–Dawley rats, rendered hydrocephalic by intra-cisternal injection of kaolin, in concentrations significantly higher than age-matched and sham-operated controls [19, 20]. In this histological and immunohistochemical study we report the effects of induced hydrocephalus on a transgenic rat model of AD (tgAPP21): a double transgenic (*Sw/Ind* mutant) human amyloid precursor protein (APP) construct that expresses high levels of human APP and Aβ40 [21]. Our hypothesis was that if the CSF was not an important clearance pathway for Aβ removal from the brain, then the accumulation of Aβ would be the same between hydrocephalic and control tgAPP21 rats. We found that the hydrocephalic tgAPP21 rats accumulated Aβ40 and oligomeric Aβ, as well as manifesting evidence of vascular disease and ischemic infarction, well before age-matched and sham-operated controls. Cortical infarction was seen only in the hydrocephalic rats.

## Methods

### Animals

Breeding pairs of APP 21 transgenic rats (tgAPP21) were obtained from the Department of Veterinary Pathobiology at the University of Missouri. These rats express high levels of human APP and naturally overproduce Aβ40, but not Aβ42. The tgAPP21 rats were produced from inbred Fischer 344 rats that express human APP driven by the ubiquitin-C promoter. They were generated via lentiviral vector infection of the Fischer 344 zygotes [21]. Immunohistochemistry in brain showed that the human APP transgene was expressed in neurons, but not in glial cells. After quarantine, the tgAPP21 rats were allowed to breed normally. The rats were housed in the veterinary care facility of the Aldrich Laboratories at Rhode Island Hospital and had food and water *ad lib.* All experiments were approved by the Institutional Animal Care and Use Committee (IACUC) at Rhode Island Hospital.

Hydrocephalus was induced by cisternal injection of kaolin (aluminum silicate 0.9%). The technique has been previously published [19, 20, 22]. Thirty-five tgAPP21 rats were used in these studies. Six-month (n = 7) and twelve-month-old (n = 9) rats had hydrocephalus induced by cisternal kaolin injection. After 10 weeks or six months of hydrocephalus the rats were euthanized by intra-peritoneal pentobarbital injection (125 mg/kg). Age-matched naïve and sham-operated tgAPP21 rats served as controls (n = 19) and their brains were processed in exactly the same way. Three of the 12-month-old hydrocephalic rats and four of the controls were allowed to survive to a natural death to assess the effects of the vascular changes on brain parenchyma (see Table 1).

**Table 1:** **Summary of experimental animals and histological findings**

Duration	Group	N	Ventricular Enlargement	Histiocytic & Granulomatous Inflammation	Neuronal Aβ40	Neuronal Aβ42	Neuronal Oligomeric Aβ	Vascular Pathology	Ischemic Infarcts
6 mo. + 10 wk.	Kaolin	4	**++**	**+++**	**++**	**-**	**++**	**++**	**-**
6 mo. + 10 wk.	Sham	2	**-**	**-**	**+**	**-**	**+**	**+**	**-**
6 mo. + 10 wk.	Naïve	2	**-**	**-**	**+**	**-**	**+**	**+**	**-**
6 mo. + 6 mo.	Kaolin	3	**+**	**+**	**++**	**-**	**+++**	**++**	**-**
6 mo. + 6 mo.	Sham	2	**-**	**-**	**+**	**-**	**++**	**+**	**-**
6 mo. + 6 mo.	Naïve	2	**-**	**-**	**+**	**-**	**++**	**+**	**-**
12 mo. + 10 wk.	Kaolin	4	**++**	**+++**	**++**	**-**	**+++**	**+++**	**-**
12 mo. + 10 wk.	Sham	2	**-**	**-**	**+**	**-**	**++**	**+**	**-**
12 mo. + 10 wk.	Naïve	2	**-**	**-**	**+**	**-**	**++**	**+**	**-**
12 mo. + 6 mo.	Kaolin	2	**+**	**+**	**+++**	**-**	**+++**	**+++**	**+**
12 mo. + 6 mo.	Sham	2	**-**	**-**	**+**	**-**	**++**	**+**	**-**
12 mo. + 6 mo.	Naïve	1	**-**	**-**	**+**	**-**	**++**	**+**	**-**
12 mo. to natural end-of-life	Kaolin	3	**+**	**+**	**+++**	**-**	**+++**	**+++**	**++**
12 mo. to natural end-of-life	Sham	2	**-**	**-**	**+**	**-**	**++**	**++**	**-**
12 mo. to natural end-of-life	Naïve	2	**-**	**-**	**+**	**-**	**++**	**++**	**-**

The pathology and morphological changes, as observed in histochemically-stained sections, were qualitatively graded using a scale ranging from no detectable changes (−) to mild (+), moderate (++), or severe (+++) changes.

After intracardiac cannulation and perfusion with phosphate-buffered saline, the brains were removed and immersed in 4% paraformaldehyde. Following standard tissue processing and paraffin embedding procedures, coronal brain sections were serially cut at 8 μm starting from the level of the median eminence. Ventricular enlargement was measured by the Evans ratio for control rats compared to rats at 10 weeks post hydrocephalus induction. The maximum ventricular diameter on coronal section at the bregma was divided by the maximum brain diameter on the post-mortem brain sections.

We analyzed Aβ burden by immunohistochemistry (Aβ40, Aβ42 and oligomeric Aβ), and vascular integrity by histochemical staining (Masson trichrome and Verhoeff-Van Gieson) at 10 weeks (n = 8) and six months (n = 5) post hydrocephalus induction. We also analyzed whether the vascular pathology seen in tgAPP21 rats, which normally develop amyloid angiopathy, was accelerated by hydrocephalus. Age-matched naïve and sham-operated tgAPP21 rats served as controls (n = 15).

### Immunohistochemistry

Eight μm-thick tissue sections (on poly-L-lysine-coated slides) were incubated in an oven at 60°C for 1 hour, and after deparaffinization and rehydration, sections were treated with hot (85°C) 10 mM citrate buffer, pH 6, for 15 minutes. Sections were washed with distilled water and then quenched with a dual endogenous enzyme-blocking reagent (Dako, Carpinteria, CA, USA; Catalog #S2003) for 10 minutes at room temperature to eliminate endogenous peroxidase activity. After washing in 0.05 M Tris-buffered saline with 0.05% Tween-20 (TBST), pH 7.6, sections were incubated overnight at 4°C with rabbit polyclonal antibodies directed against Aβ40 (Alpha Diagnostic International, San Antonio, TX, USA; Catalog #BAM401-A, diluted 1:100), Aβ42 (Alpha Diagnostic International; Catalog #BAM421-A, diluted 1:200), or oligomeric Aβ (A11; Chemicon, Temecula, CA, USA; Catalog #AB9234, diluted 1:2000). After washing the sections in TBST, a horseradish peroxidase (HRP)-labeled polymer conjugated with secondary antibodies (anti-rabbit; Dako; Catalog #K4002) was applied for 30 minutes at room temperature, in accordance with the EnVision + System for immunohistochemical staining. The tissue sections were washed in TBST and then the immunoreaction product was developed using 3,3-diaminobenzidine (Dako; Catalog #K3468) as the chromogen. Sections were dehydrated through a series of graded alcohols back to xylene, and then coverslipped and sealed using Cytoseal XYL (Richard-Allan Scientific, Kalamazoo, MI, USA; Catalog #8312-4). Primary antibody omission controls were run alongside the other samples to check for non-specific binding due to the secondary antibodies, and advanced AD human prefrontal cortical sections were run as positive controls. In place of using a counterstain on immunohistochemically-stained slides, adjacent serial sections were stained with hematoxylin and eosin (H&E) for analysis of general tissue morphology.

### Immunofluorescence

Following deparaffinization and rehydration, tissue sections were treated with hot (85°C) 10 mM citrate buffer, pH 6, for 15 minutes. Sections were washed with distilled water and then quenched with a dual endogenous enzyme-blocking reagent (Dako) for 10 minutes at room temperature. After washing in TBST, sections were blocked with 5% normal goat serum (Vector Laboratories, Burlingame, CA, USA; Catalog #S-1000) for 2 hours at room temperature, and then dually incubated overnight (at 4°C) with the following primary antibodies: a mouse monoclonal antibody directed against NeuN (A60; Abcam, Cambridge, MA, USA; Catalog #ab77315, diluted 1:1000) and a rabbit polyclonal antibody directed against oligomeric Aβ (A11; Chemicon, diluted 1:2000). Sections were then washed in TBST, and the secondary antibodies were applied for 90 minutes at room temperature in the dark: AlexaFluor 488 goat anti-mouse IgG (Molecular Probes, Eugene, OR, USA; Catalog #A-11001, diluted 1:1000) and AlexaFluor 594 goat anti-rabbit IgG (Molecular Probes; Catalog #A-11012, diluted 1:1000). To eliminate possible lipofuscin autofluorescence, tissue sections were incubated in a 0.3% Sudan Black B (Sigma-Aldrich, St. Louis, MO, USA; Catalog #S-0395) solution in 70% ethanol for 20 minutes at room temperature in the dark. Sections were washed in distilled water and coverslipped using Vectashield Hard Set Mounting Medium with DAPI (Vector Laboratories; Catalog #H-1500). Primary antibody omission controls were run alongside the other samples to check for non-specific binding due to the secondary antibodies, and advanced AD human prefrontal cortical sections were run as positive controls.

### Masson trichrome staining

Masson trichrome staining was carried out in accordance with well-characterized protocols [23, 24]. Briefly, tissue sections were deparaffinized and hydrated in distilled water prior to a 1-hour treatment in Bouin’s fixative (Richard-Allan Scientific; Catalog #NC9674780) at 56°C. Sections were washed in running distilled water until clear, and then stained in Weigert’s iron hematoxylin (Richard-Allan Scientific; Catalog #NC9231529) for 10 minutes. Following a 10-minute wash in running water, sections were stained in Biebrich scarlet-acid fuchsin (Richard-Allan Scientific; Catalog #NC9424144) for 2 minutes. Sections were rinsed in distilled water followed by a 10-minute differentiation in phosphomolybdic-phosphotungstic acid (Richard-Allan Scientific; Catalog #NC9443038). Aniline blue (Richard-Allan Scientific; Catalog #NC9684104) was used as a counterstain for 10 minutes, and then sections were differentiated in 1% acetic acid for 3 minutes. Sections were dehydrated through a series of graded alcohols back to xylene, and then coverslipped and sealed using Cytoseal XYL (Richard-Allan Scientific).

### Verhoeff-Van Gieson staining

The Verhoeff-Van Gieson staining protocol for elastic fibers was performed using well-established protocols [24, 25]. Briefly, tissue sections were deparaffinized and hydrated to distilled water followed by a1-hour incubation in Verhoeff’s working solution (Polysciences, Warrington, PA, USA; Catalog #25089). Sections were rinsed in running water, and then differentiated in 2% ferric chloride (Sigma-Aldrich; Catalog #451649) for 2 minutes. Following a 10-minute wash in running water, sections were treated with 5% aqueous sodium thiosulfate (Sigma-Aldrich; Catalog #S7026) for 1 minute. Tissue sections were then washed in running water for 5 minutes, and counterstained with Van Gieson’s solution (Poly Scientific, Bay Shore, NY, USA; Catalog #s289) for 3 minutes. Sections were quickly dehydrated through a series of graded alcohols back to xylene, and then coverslipped and sealed using Cytoseal XYL (Richard-Allan Scientific).

### Microscopy, image acquisition & qualitative grading

All immunohistochemistry and histochemically-stained slides were converted to digital images using Aperio Scan Scope (Aperio Technologies, Vista, CA, USA) as 8-bit acquisitions of color. For confocal microscopy, images were acquired with a Nikon C1si confocal microscope (Nikon Inc., Melville, NY, USA) using 488 nm and 561 nm diode lasers. Serial optical sections were performed with EZ-C1 computer software (Nikon Inc.). Z-series sections were collected at 1.5 μm with a 20× PlanApo lens and scan zoom of 2×. Each wavelength was acquired separately by invoking frame lambda, and images were processed with Elements computer software (Nikon Inc.). Pathology and morphological changes, as observed in histochemically-stained sections, were qualitatively graded using a scale ranging from no detectable changes (−), to mild (+), moderate (++), or severe (+++) changes.

## Results

**Figure 1:**
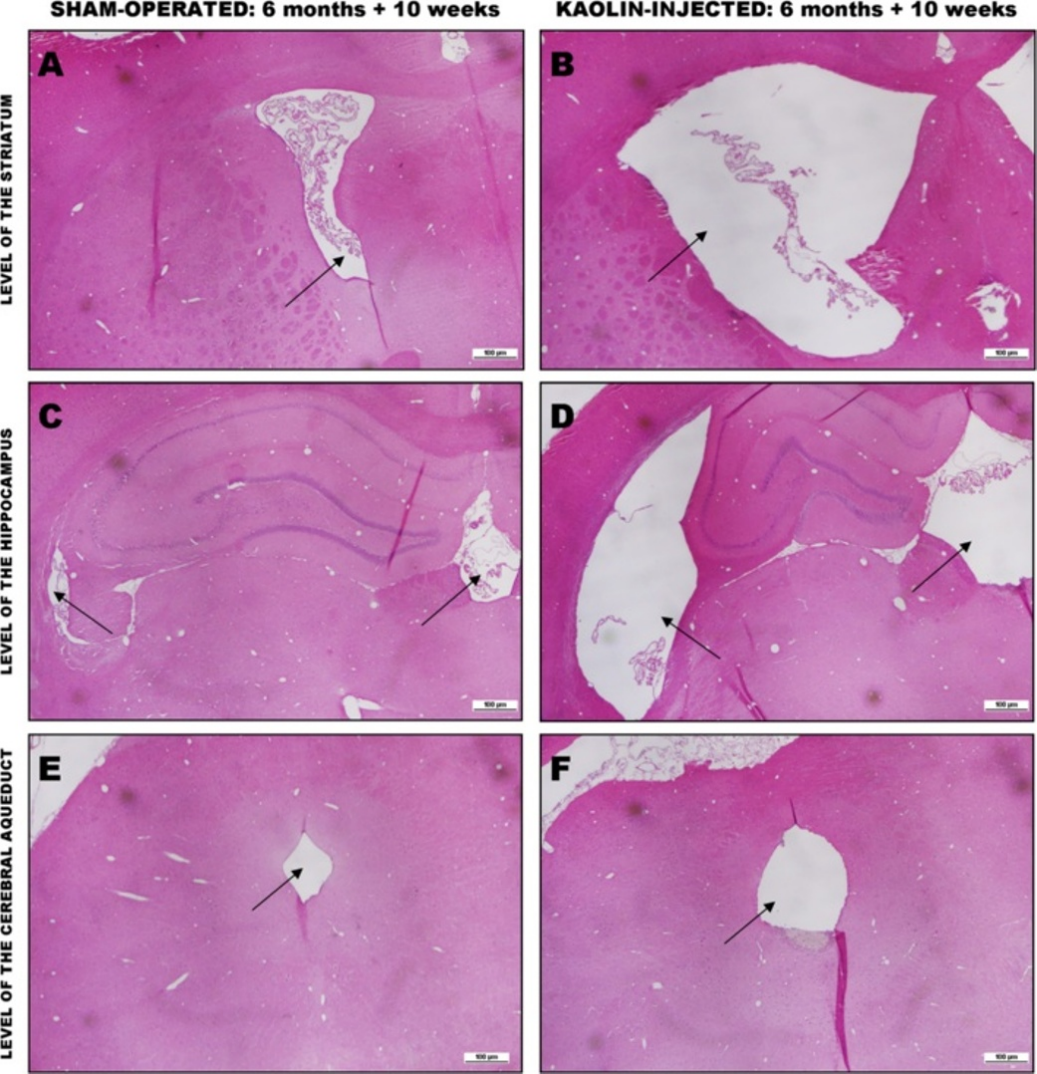
**Sections of sham-operated and kaolin-injected rats 10 weeks after injection.** Comparison of a representative sham-operated tgAPP21 rat (left panels) to a representative kaolin-injected tgAPP21rat (right panels) at three different coronal brain levels: the level of the striatum **(A & B)**, the level of the hippocampus **(C & D)**, and the level of the cerebral aqueduct **(E & F)**. Note the marked ventricular and aqueductal enlargement in the kaolin-treated tgAPP21 rat compared to sham-operated rat (arrows). H&E stain, ×10.

All histological and immunohistochemical findings are summarized in Table 1. The tgAPP21rats injected with kaolin developed hydrocephalus similar to that reported in our wild-type Sprague–Dawley rats [20]. Evans index of ventricular size in the hydrocephalic tgAPP21 rats was significantly larger than controls and was similar to what we previously reported [10 weeks 0.30 ± 0.04 compared to sham-operated controls 0.19 ± 0.02 (mean ± SD)]. Figure 1 compares the ventricular and aqueductal morphological changes observed in a typical hydrocephalic tgAPP21 rat to a typical age-matched sham-operated control.

**Figure 2:**
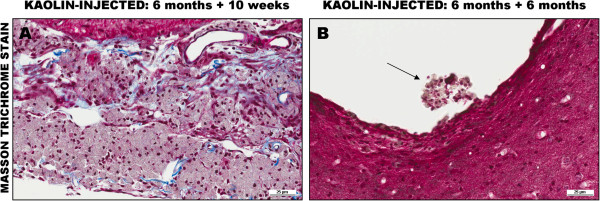
**Effects of cisternal kaolin injection. (A)** At 10 weeks post-kaolin injection, there is marked histiocytic and granulomatous inflammation composed of many macrophages filled with kaolin in the SAS at the base of the brain (here around the hypothalamus and 3rd ventricle). **(B)** At six months post-kaolin injection, the inflammation has subsided leaving scarring and blockage in the SAS (below the 3rd ventricle) and a few small collections of histiocytes in the ventricle walls, here the 3rd ventricle (arrow). Masson trichrome stain, ×200.

The hydrocephalus was due to an intense histiocytic and granulomatous reaction in the SAS which was largely resolved by six months, though some small granulomas remained. Despite the resolution of the inflammation, scarring and blockage of the SAS remained (Figure 2).

**Figure 3:**
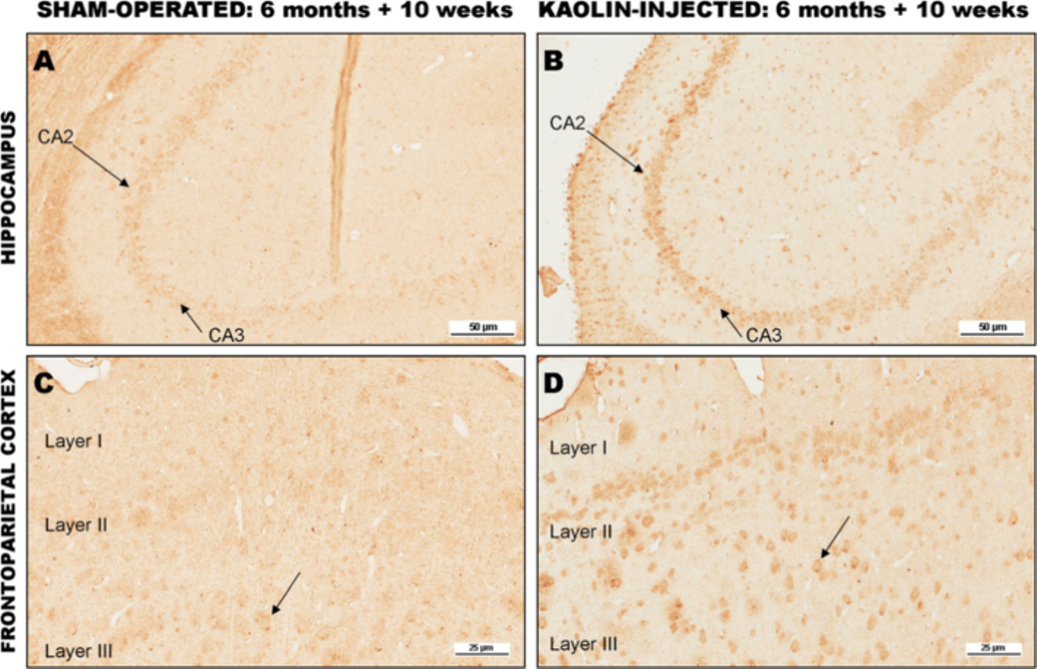
**Immunohistochemical staining for Aβ40. (A)** Hippocampal neurons in a sham-operated six-month-old tgAPP21 rat (arrows). There is minimal immunoreactivity evident 10 weeks after sham-surgery, x80. **(B)** Hippocampal neurons in a hydrocephalic six-month-old tgAPP21 rat 10 weeks after kaolin injection demonstrating enhanced immunoreactivity in areas CA2 and CA3 (arrows), ×80. **(C)** Frontoparietal cortical neurons (arrow) in a sham-operated six-month-old tgAPP21 rat at 10 weeks post-surgery showing minimal Aβ40 immunoreactivity, ×200. **(D)** There is more robust neuronal immunoreactivity in the frontoparietal cortex in six-month-old tgAPP21 rats 10 weeks following kaolin injection (arrow), ×200.

Aβ immunostaining showed a marked increase in intraneuronal Aβ40 in the hippocampus and frontoparietal cortex compared to sham-operated controls (Figure 3). No amyloid plaques were observed, but diffuse cytoplasmic Aβ40 immunoreactivity was evident across multiple neuronal populations. The absence of dense-core plaques was not surprising given the absence of Aβ42 accumulation.

**Figure 4:**
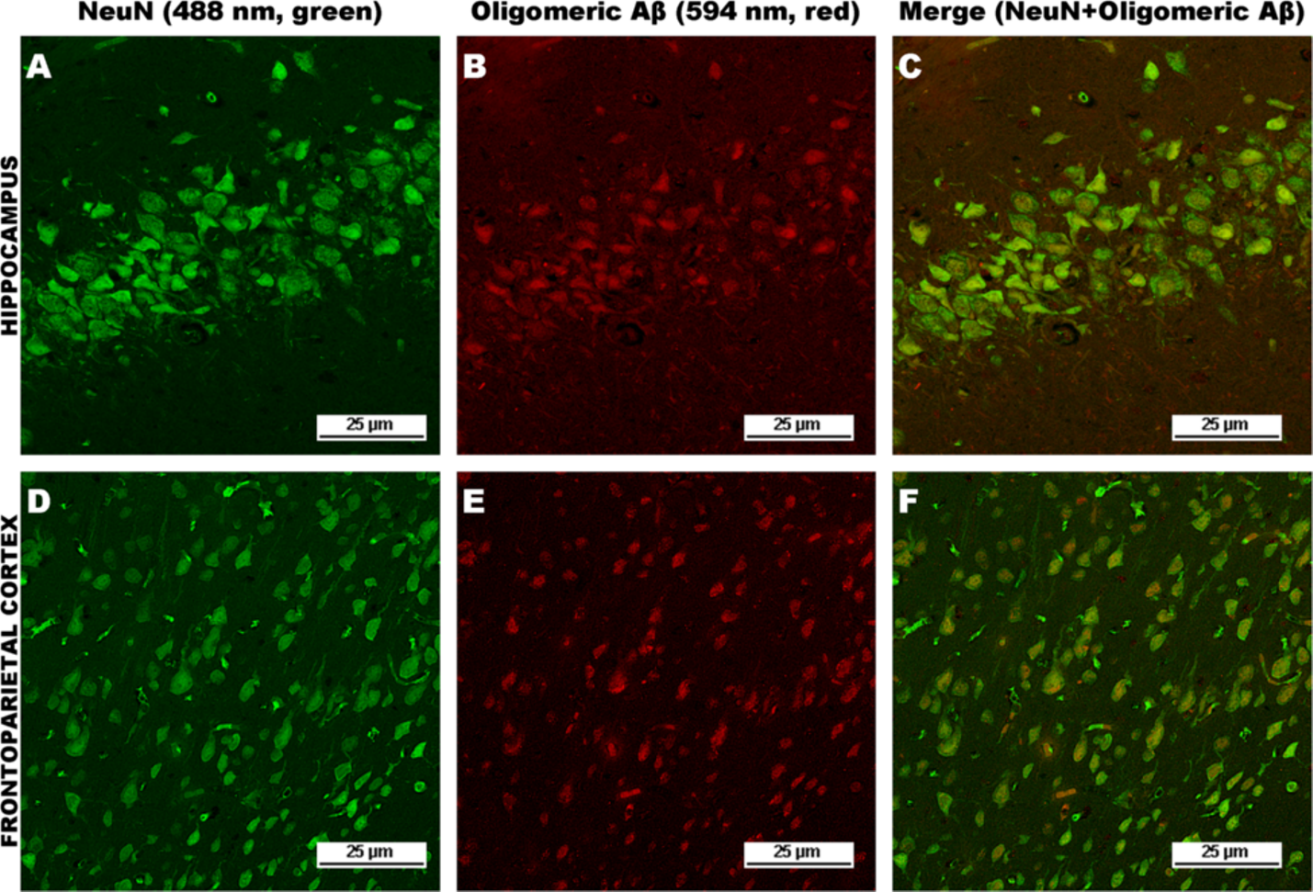
**Oligomeric Aβ immunoreactivity is intraneuronal.** Top row: hippocampal NeuN **(A)** and oligomeric Aβ **(B)** immunoreactivity in a six-month-old tgAPP21 rat at 10 weeks post-kaolin showing a strong overlap of immunopositive neuronal cell bodies **(C)**, ×400. Bottom row: Frontoparietal cortical NeuN **(D)** and oligomeric Aβ **(E)** immunoreactivity in a six-month-old tgAPP21 rat at 10 weeks post-kaolin also reveals a widespread concomitance of immunopositive neuronal cell bodies **(F)**, ×200.

Although Aβ42 is more apt to self-assemble, Aβ40 can also self-assemble into oligomeric forms. Oligomeric Aβ immunoreactivity was predominately confined to neurons in both the hippocampus and frontoparietal cortex of tgAPP21 rats (Figure 4).

**Figure 5:**
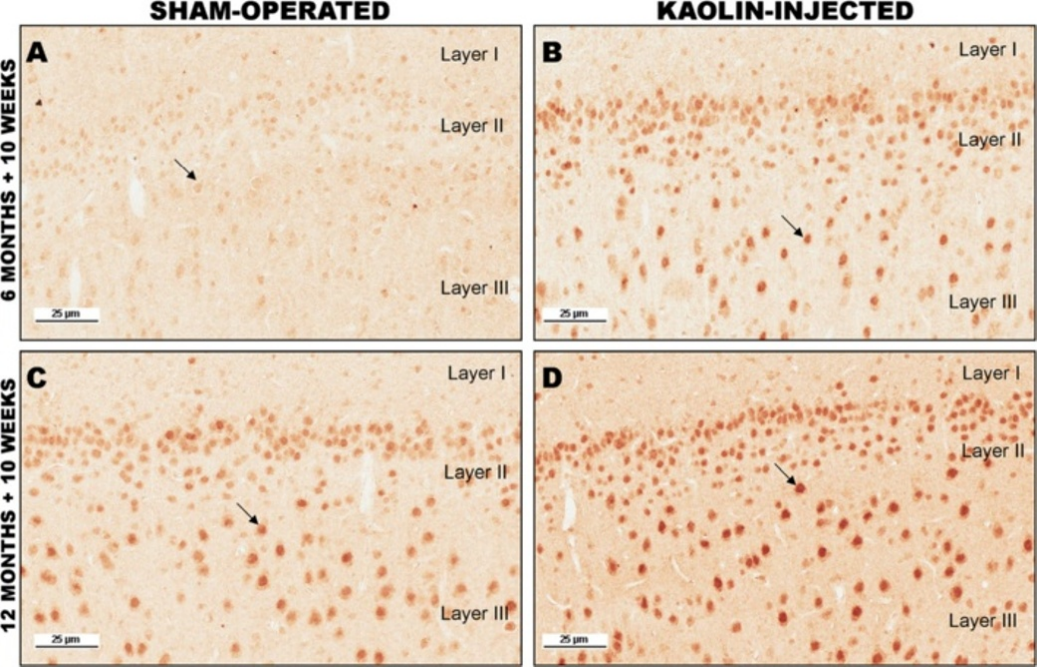
**Oligomeric Aβ in tgAPP21hydrocephalic rat frontoparietal cortex compared to control rat (arrows).** Top row: six-month-old rat. **(A)** sham-operated control brain showing minimal immunostaining for oligomeric forms, ×200. **(B)** six-month-old rat 10 weeks post-kaolin hydrocephalus, ×200. There is a marked increase in immunoreactive product. Bottom row: 12-month-old rat. **(C)** sham-operated control showing moderate immunoreactivity for oligomeric Aβ, ×200. **(D)** 12-month-old rat 10 weeks post-kaolin hydrocephalus showing a significant increase in oligomeric Aβ, ×200.

In hydrocephalic tgAPP21 rats, compared to controls, there was increased oligomeric Aβ immunoreactivity in addition to the increase in Aβ 40 in both hippocampal (data not shown) and cortical neurons at 10 weeks post-hydrocephalus induction in 6 and 12-month-old animals (Figure 5).

**Figure 6:**
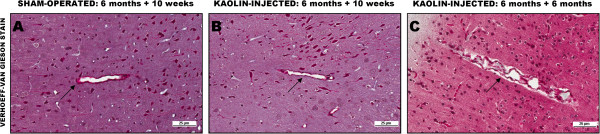
**Degenerative changes in hydrocephalic rat vessels compared to controls (arrows). (A)** Age-matched sham-operated control demonstrating a normal appearing parenchymal vessel. **(B)** six-month-old tgAPP21 rat at 10 weeks post-kaolin showing very subtle vascular changes. **(C)** six-month-old tgAPP21 rat six months post hydrocephalus induction demonstrating rather marked vessel wall degenerative changes and expansion of the Virchow-Robin space. Verhoeff-Van Gieson stain, ×200.

Vascular pathology was accelerated by the induction of hydrocephalus compared to controls. In the six-month-old rats, subtle degenerative changes were noted in vessel walls at 10 weeks post-kaolin, whereas in the six-month-old rat at six months post-kaolin and in the 12-month-old rat 10 weeks post-kaolin, more pronounced degenerative changes were seen with clear expansion of the Virchow-Robin space in interstitial vessels (Figure 6).

**Figure 7:**
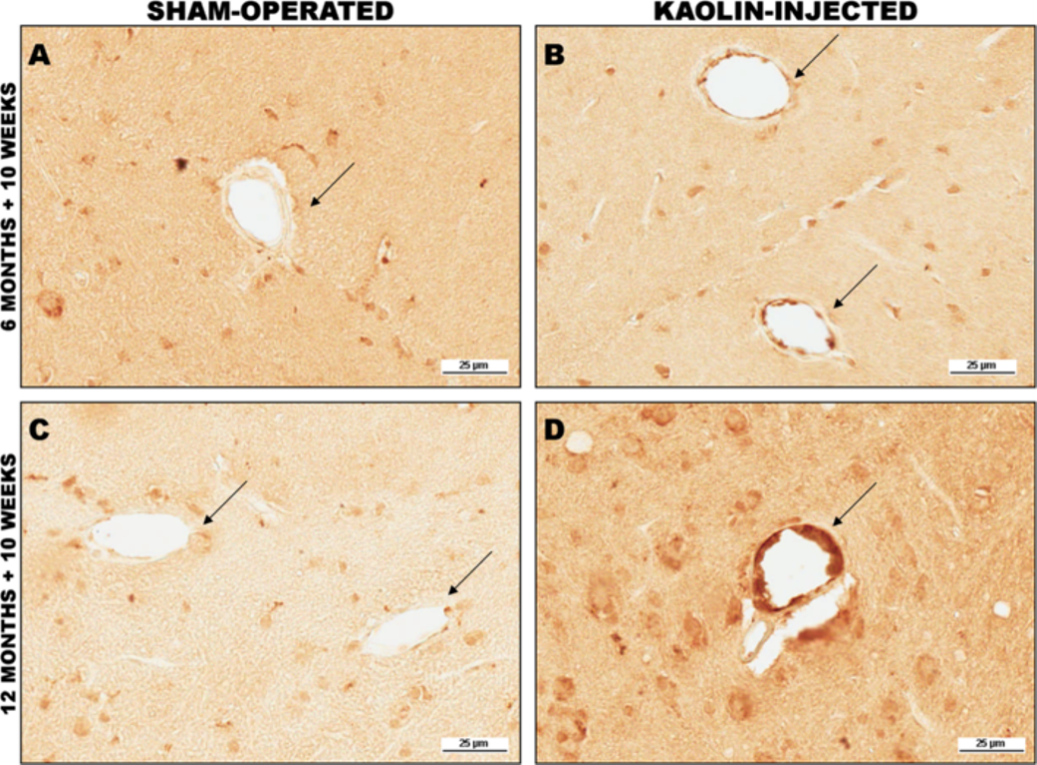
**Deposition of Aβ40 in brain parenchymal vessels (arrows).** Top row: six-month-old tgAPP21 rat. **(A)** sham-operated age-matched control, x200. **(B)** Hydrocephalic tgAPP21 rat 10 weeks post kaolin injection, ×200. Note a moderate increase in immunoreactivity in the vessels of the hydrocephalic rat compared to control. Bottom row: 12-month-old tgAPP21 rat. **(C)** Age-matched, sham-operated control showing minimal vascular Aβ immunostaining, x200. **(D)** 12-month-old tgAPP21 rat 10 weeks after hydrocephalus induction showing marked deposition of Aβ in vessel walls, ×200.

Amyloid angiopathic changes were seen by immunostaining for Aβ40. These changes were more dramatic in the 12-month-old tgAPP21 rats than in the six-month-old rats, and in both sets of hydrocephalic rats, the difference from sham-operated controls was clearly evident (Figure 7).

**Figure 8:**
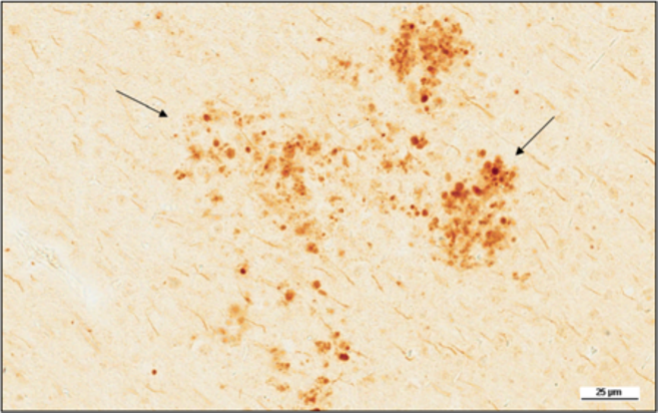
**Evidence of cortical infarcts in hydrocephalic tgAPP21 rats.** Frontoparietal cortex stained for Aβ40. Arrows point to areas of infarction, ×200.

Seven of the tgAPP21 rats were allowed to reach their natural end of life (approximately 30 months). Three hydrocephalic rats were compared to four controls. The hydrocephalic tgAPP21 rats were found to have frequent areas of microscopic cortical infarction in their brains (Figure 8). In the non-kaolin controls, no infarcts were seen.

## Discussion

Clearance of macromolecules, such as Aβ, from the brain interstitial space involves at least four different pathways: i) via *in situ* degradation [26–30], ii) active transport across the blood–brain barrier [31–36], iii) across the choroid plexus epithelium by active transport [37], and iv) via the production and turnover of the CSF. CSF turnover is defined as the number of times the CSF is renewed in 24 hours and is calculated by dividing CSF production in 24 hours by the volume of the CSF space [38–42]. Normally in humans, CSF turnover occurs 4–5 times per day.

Hydrocephalus is known to disrupt normal CSF physiological functions. In both AD and in hydrocephalic patients, CSF turnover is reduced threefold [6, 43, 44]. In both wild-type rat models of NPH and in human NPH patients therefore, CSF clearance of potentially toxic solutes like Aβ is significantly reduced, resulting in the accumulation of these molecules in brain parenchyma. Several investigations in laboratory animals have described significantly decreased CSF production and turnover after kaolin hydrocephalus induction [45, 46], and is also seen in humans with NPH [44]. Resistance to CSF absorption is also increased in hydrocephalus [47, 48]. Despite eventual clearance of the inflammation produced by the kaolin, increased resistance to CSF absorption and decreased compliance remain [47, 48].

This study examined the effects of kaolin-induced hydrocephalus on amyloid accumulation and vascular pathology in a transgenic rat model of AD. The analysis was carried out by qualitative histological and immunohistochemical staining, comparing the hydrocephalic tgAPP21 brains to age-matched, sham-operated and naïve controls. We found that induced hydrocephalus accelerated Aβ accumulation in neurons and Aβ deposition in the cerebral vasculature, presumably due to decreased clearance of Aβ. Aβ immunostaining in cerebral cortex and hippocampus was increased in the hydrocephalic rats compared to controls, and amyloid angiopathic degeneration of cerebral vessels was also accelerated compared to controls. The amyloid angiopathy associated with the hydrocephalic rats appeared to cause microscopic ischemic infarcts not seen in the control animals.

It is well known that there is variability in the degree of hydrocephalus produced by intracisternal kaolin. Also in any qualitative histological and immunohistochemical study, fixation artifact must always be considered in assessing changes. Therefore, comparison to both sham-operated and naïve controls processed in exactly the same way as the hydrocephalic rats, is essential to identifying true differences from artifact. The microscopic findings in the two groups (hydrocephalic and controls) in this study were internally consistent but were strikingly different in Aβ accumulation, self-assembly into oligomeric forms and vascular pathology. Although not quantitative, the group comparisons were sufficiently different to conclude that accelerated amyloid deposition and vascular pathology occurs in tgAPP21 rats with kaolin-induced hydrocephalus.

One can argue that the tgAPP21 rat is more a model for amyloid angiopathy [49] rather than AD, in that there is no increase in Aβ42 concentrations and no amyloid plaque formation was evident. Instead we see that the predominantly expressed Aβ40 accumulates in the cerebral microvessels, reportedly localized to the basement membrane [50, 51]. However, our study was meant to explore whether the CSF plays a significant role in the clearance of macromolecular solutes from the brain interstitial space, and whether its failure in chronic hydrocephalus accelerates the accumulation of many brain metabolites. The present study suggests that this is the case in rats and likely in humans as well.

## Conclusions

The results of this study underscore the importance of normal CSF physiologic functions in clearing potentially toxic macromolecules from the brain. The study shows that kaolin-induced hydrocephalus can accelerate intraneuronal Aβ accumulation and self-assembly, and accelerate vascular pathology in tgAPP21 rats. In addition, it demonstrates that disrupted CSF production and turnover results in impaired Aβ clearance from the brain and accelerates vascular pathology in chronic hydrocephalus. The high co-morbidity seen in NPH, AD and CVD is likely not an age-related coincidence, but rather a convergence of pathologies related to reduced solute clearance.

## Abbreviations

Aβ: Amyloid-beta peptide
AD: Alzheimer’s disease
APP: Amyloid precursor protein
CSF: Cerebrospinal fluid
CVD: Cerebrovascular disease
H&E: Hematoxylin and eosin
HRP: Horseradish peroxidase
IACUC: Institutional Animal Care and Use Committee
NPH: Normal pressure hydrocephalus
pTau: Hyperphosphorylated tau protein
REM: Rapid eye movement
SAS: Subarachnoid space
TBST: 0.05 M Tris-buffered saline with 0.05% Tween-20
tgAPP21: Double mutant APP_*Sw/Ind*_ transgenic rat line.
